# Interleukin-6 for early diagnosis of neonatal sepsis with premature rupture of the membranes: A meta-analysis

**DOI:** 10.1097/MD.0000000000013146

**Published:** 2018-11-21

**Authors:** Xia Qiu, Li Zhang, Yu Tong, Yi Qu, Huiqing Wang, Dezhi Mu

**Affiliations:** a Department of Pediatrics, West China Second University Hospital; b Key Laboratory of Obstetric & Gynecologic and Pediatric Diseases and Birth Defects of Ministry of Education, Sichuan University, Chengdu, China.

**Keywords:** interleukin-6, meta-analysis, neonatal sepsis, premature rupture of the membranes

## Abstract

**Background:**

Premature rupture of the membranes (PROM) is the principal risk factor for neonatal sepsis. Interleukin-6 (IL-6) has been investigated for early diagnosis of neonatal sepsis, but not for diagnosis of neonatal sepsis with PROM. The objective of this study is to investigate the early diagnostic value of IL-6 for neonatal sepsis with PROM.

**Methods:**

The literature was searched using PubMed, Embase, Cochrane Library, Web of Science, CNKI, Wan Fang, VIP, and CBM databases until March 2018. Each study was evaluated using Quality Assessment of Diagnostic Accuracy Studies tool-2. We used a bivariate diagnostic random-effects model.

**Results:**

The overall pooled sensitivity, specificity, positive likelihood rate, negative likelihood rate, diagnostic odds ratio, and area under the summary receiver operating characteristic curve were 0.85 (95% confidence interval [CI]: 0.81–0.91), 0.88 (95% CI: 0.86–0.91), 9.94 (95% CI: 4.27–23.15), 0.14 (95% CI: 0.06–0.32), 79.26 (95% CI: 23.42–268.26), and 0.9473, respectively, which showed high accuracy in diagnosing neonatal sepsis with PROM. The types of sepsis might be connected with the source of heterogeneity (*P = *.0351).

**Conclusion:**

IL-6 is therefore a sensitive and specific diagnostic marker for the early diagnosis of neonatal sepsis with PROM.

## 1 Introduction

Premature rupture of the membranes (PROM) is membrane rupture before the onset of uterine contractions; the incidence of PROM is 10 per 100 pregnancies.^[1]^ Preterm premature PROM (PPROM) is defined as PROM prior to 37 weeks of gestation, which occurs in 3 of 100 pregnancies.^[2]^ PROM is associated with several neonatal diseases, including neonatal infection, necrotizing enterocolitis, and intraventricular hemorrhage.^[3–5]^

Neonatal sepsis is a serious systemic disease affecting infants in the first month of life.^[6]^ PPROM is the principal risk factor for neonatal sepsis, which is associated with increased fetal infection morbidity and mortality in the neonatal period.^[7–13]^ In recent years, although the management of newborns has improved, the incidence of neonatal sepsis is 1 to 10 per 1000 live births.^[14]^ Neonatal sepsis in PROM is easily misdiagnosed because the early clinical signs are nonspecific and variable.^[15]^ A positive blood culture is the reference standard for detecting neonatal sepsis with PROM. However, the blood culture results require at least 24 to 72 hours, and reveals positivity in 19.2% of cases, which is lowly sensitive.^[16,17]^ It has various practical limitations. Routine laboratory tests, such as C-reactive protein (CRP) and procalcitonin (PCT), which mediated the host response to bacterial infection and released in the neonatal blood, have low sensitivity.^[18,19]^ In addition, evidence indicates that antibiotic prophylaxis applied in 42% of the deliveries showed no beneficial effect on the incidence of neonatal sepsis and even made early diagnosis of neonatal sepsis more difficult.^[20,21]^ Therefore, early detection of neonatal sepsis with PROM should be developed to reduce the inadvertent use of antibiotics, cost of treatment, and overtreatment.

Interleukin-6 (IL-6) is a pleiotropic cytokine expressed by different cells in response to infections.^[22]^ Recently, IL-6 has been investigated for its validity in diagnosing neonatal sepsis.^[23]^ However, there are no large-scale multicenter studies or meta-analyses on IL-6 for the diagnosis of neonatal sepsis with PROM. Therefore, we conducted a meta-analysis to systematically assess all published studies on the diagnostic performance of IL-6 for detecting neonatal sepsis with PROM.

## 2 Methods

### 2.1 Literature search

The study was conducted according to the Preferred Reporting Items for Systematic Reviews and Meta-Analyses criteria.^[24]^ A computer-aided literature search was performed using PubMed, Embase, the Cochrane Library, Web of Science, CNKI, WanFang, VIP, and CBM databases for relevant citations up to March 2018 without language restrictions. Our search terms were “neonate,” “newborn,” “infant,” “sepsis,” “septicemia,” “premature rupture of membranes,” “PROM,” “interleukin-6,” and “IL-6.” The search strategy was as follows: (“interleukin-6” OR “IL-6”) AND (“neonate” OR “newborn” OR “infant”) AND (“sepsis” OR “septicemia”) AND (“premature rupture of membranes” OR “PROM”). We (XQ and LZ) also manually searched the references of the included studies and relevant reviews.

### 2.2 Literature selection

Obtained studies from the literature were reviewed independently by 2 investigators (XQ and LZ) to ensure high accuracy. Inclusion criteria for this study were as follows: assessment of the diagnostic accuracy of IL-6 for neonatal sepsis in PROM; reporting on neonatal sepsis with PROM (population); provision of IL-6 in umbilical cord blood and neonatal peripheral blood as the index test and gold standard for blood culture (index test and reference standard); inclusion of sensitivity, specificity, or sufficient information to construct the 2 × 2 tables (outcome). For studies that were published more than once, only the most recent and comprehensive report was included. Reviews, letters, case reports, animal experiments, systematic reviews, and meta-analyses were excluded from the analysis.

### 2.3 Data extraction

Two investigators (XQ and LZ) independently extracted data from selected articles. The following data were recorded: author, year of publication, regions, study population, gestational age (week), sample size, specimen, assay method, cut-off value, diagnostic gold standard, type of sepsis, sensitivity, specificity, true positive (TP), false positive (FP), false negative (FN), and true negative (TN) of IL-6. The analyses were based on previous published studies. Therefore, no patient consent, ethical approval, and institutional review board are required.

### 2.4 Quality assessment

The methodological quality of the included studies was assessed using Quality Assessment of Diagnostic Accuracy Studies tool-2 (QUADAS-2).^[25]^ Two reviewers (XQ and LZ) independently performed the quality assessment. Four domains (patient selection, index test, reference standard, and flow and timing) were evaluated for the risk of bias, and 3 domains (patient selection, index test, and reference standard) were evaluated based on applicability. Participant spectrum bias and selection bias were determined. Information bias existed for the index test. Partial verification bias, differential verification bias, and disease progression bias were related to the reference standard. Excluded data bias existed for flow and timing.^[25]^ Signaling questions were asked to help estimate the risk of bias.^[25]^ In case of unresolved disagreement, a third reviewer (YT) was consulted.

### 2.5 Statistical analysis

Meta-Disc software (version 1.4) was used to perform the statistical analysis.^[26]^ The Spearman correlation analysis was used to assess the threshold effect, and *P < *.05 was used to indicate a significant threshold effect. We further analyzed the influence of the cut-off used in each study by subgroup analysis. A bivariate random effects model was used to calculate the pooled sensitivity, specificity, positive likelihood ratio (PLR), negative likelihood ratio (NLR), and diagnostic odds ratio (DOR).^[27–28]^ Based on sensitivity and specificity, we further constructed the summary receiver operating characteristic (SROC) curve. The area under the curve (AUC) was also calculated to evaluate the diagnostic performance of IP-10; a value close to 1 indicated that IP-10 is a good diagnostic tool.^[29–31]^ Heterogeneity among the included studies was evaluated using the Cochrane *Q* test and *I*^2^ statistic. ^[32]^ Additionally, we conducted a meta-regression analysis to explore possible sources of heterogeneity. We used Deeks’ test to assess publication bias. Stata software (version 14.0) was used to draw funnel plots for analysis of publication bias.

## 3 Results

### 3.1 Literature research

**Figure 1:**
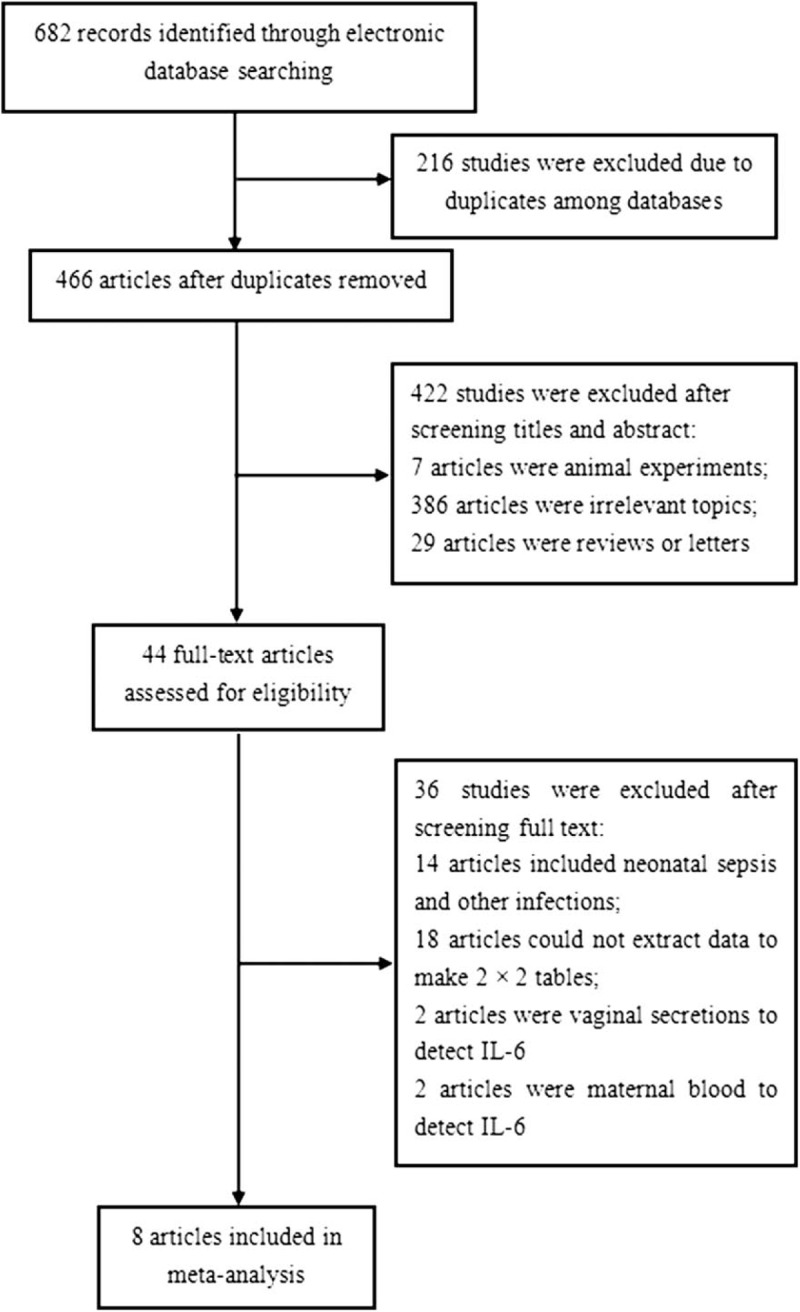
Flowchart of the process of the identified and included articles.

As shown in Figure 1, 682 literature citations were identified from database searches (PubMed: 147; Embase: 109; Cochrane Library: 9; Web of Science: 278; CNKI: 25; WanFang: 56; VIP: 8; CBM: 50). First, 216 duplicate studies were removed. Then, by reviewing the titles and abstracts, 422 articles that did not meet the inclusion criteria were excluded. After screening the full texts of 36 articles, 14 articles included other infections (pneumonia, necrotizing enterocolitis, etc.) were excluded, 18 articles which did not report related data (sensitivity, specificity, etc.) were excluded, and *2* articles detecting vaginal secretion and *2* articles detecting maternal blood were excluded. Ultimately, 8 articles^[7–11,2,12–13]^ including 9 trials met the inclusion criteria and were included in this meta-analysis.

### 3.2 Characteristics of the included studies

The characteristics of 8 eligible articles totaling 9 clinical trials, including 9 trials, are listed in Table 1.^[7–11,2,12–13]^ Seven trials were published in English while *2* were in Chinese.^[7,8]^ The publication year ranged from 2005 to 2017. Three trials were in Europe, and 6 trials were in Asia. There were 694 participants including 752 samples involved in this meta-analysis. The sensitivity, specificity, TP, FP, FN, and TN of IL-6 in each trial are shown in Table 1. Eight (89%) of studies included neonatal sepsis with PPROM and healthy neonates with PPROM, and only one study included neonatal sepsis with PROM and healthy neonates with PROM.^[8]^ Only one study did not report patient's age.^[11]^ Patients’ age, number of patients, assay method, specimen, cut-off value, type of sepsis sensitivity, specificity, TP, FP, FN, and TN of IL-6 in each trial are shown in Table 1.

**Table 1:**
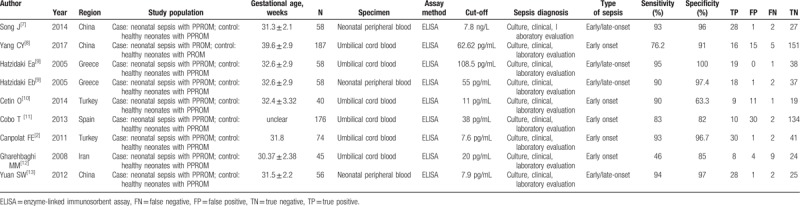
Main characteristics of studies included in the meta-analysis.

### 3.3 Quality assessment

**Figure 2:**
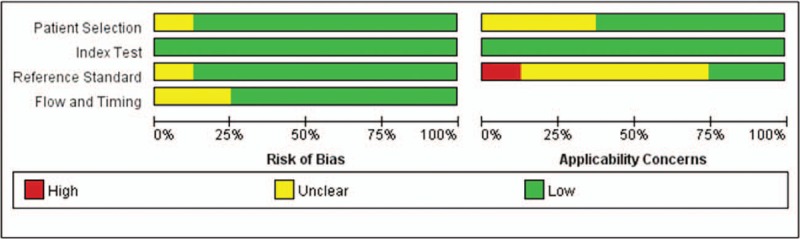
Risk of bias and applicability concerns of included studies.

The QUADAS-2 was used to assess the methodological quality of the included articles (Fig. 2). Patient selection showed low bias in 7studies and unclear bias in one study. All studies had low bias in their index tests. Seven studies were allocated as having low bias in their reference standard, and one study showed unclear bias. Six studies had low bias in flow and timing, and *2* studies showed unclear bias. Regarding applicability concerns, 5 studies showed low concerns in patient selection, and 3 studies showed unclear concerns. All studies had applicability concerns as low concerns in the index tests; *2* studies were allocated as low concern in the reference standard, one study showed high concerns, and 5 studies showed unclear concern.

### 3.4 Pooled analysis

**Figure 3:**
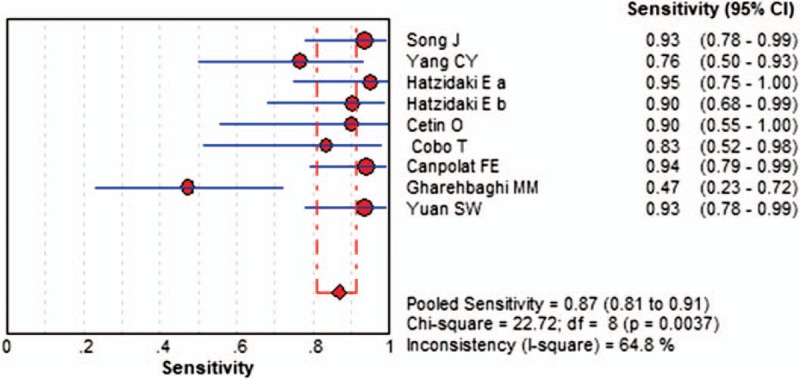
The forest plots of the pooled sensitivity of IL-6 to diagnose neonatal sepsis in PROM. CI = confidence interval, IL-6 = interleukin-6, PROM = premature rupture of the membranes.

**Figure 4:**
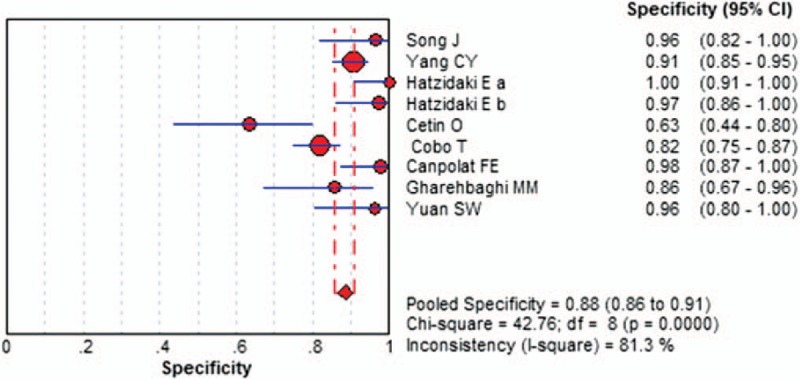
The forest plots of the pooled specificity of IL-6 to diagnose neonatal sepsis in PROM. CI = confidence interval, IL-6 = interleukin-6, PROM = premature rupture of the membranes.

**Figure 5:**
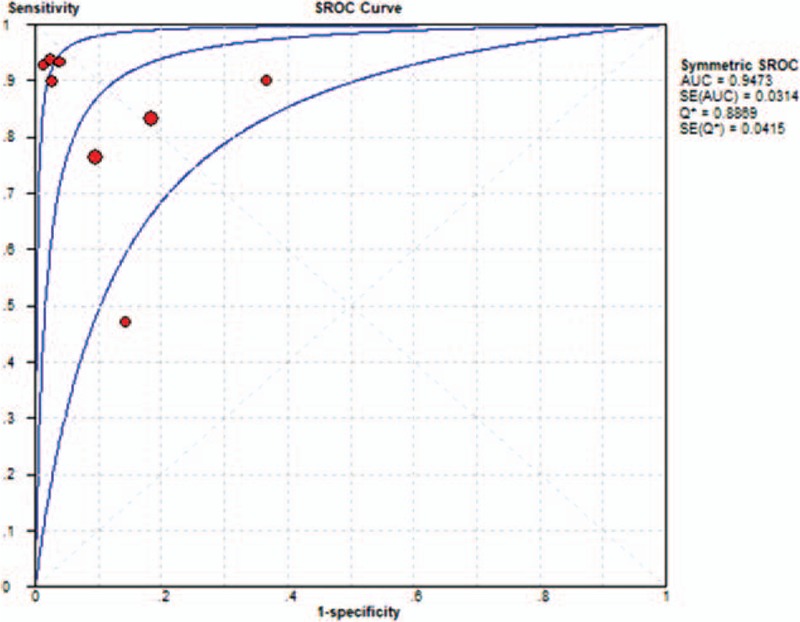
The summary receiver operating characteristic curve for assessment of IL-6 to diagnose neonatal sepsis in PROM. AUC = area under curve, SROC = summary receiver operating characteristic, IL-6 = interleukin-6, PROM = premature rupture of the membranes, SE = standard error.

The Spearman correlation coefficient was −0.613, and the *P*-value was .079, and there was no threshold effect; the DOR also showed a nonthreshold effect (Cochran-*Q* = 26.69, *I*^2^ = 70%, *P = *.0008). We used a random-effects model to detect IL-6 for neonatal sepsis with PROM. The sensitivity ranged from 0.47 to 0.95 (pooled sensitivity: 0.87, 95% CI: 0.81–0.91), whereas specificity ranged from 0.63 to 1.00 (pooled specificity: 0.88, 95% CI: 0.86–0.91) (Figs. 3 and 4). The pooled PLR of IL-6 was 9.94 (95% CI: 4.27–23.15), and the pooled NLR was 0.14 (95% CI: 0.06–0.32). In addition, the pooled DOR was 79.26 (95% CI: 23.42–268.26). DOR, which could be used to assess the accuracy of the test, showed that the discriminate effect in our study was good. For all aforementioned effect sizes, significant heterogeneities were observed (*I*^2^ > 50%). From the SROC in Figure 5, the AUC was 0.9473 with a standard error of 0.0314, which represented perfect discriminatory ability.

### 3.5 Sensitivity analysis

Excluding the study that showed high concerns in reference standard,^[9]^ the pooled sensitivity, specificity, and AUC of IL-6 for neonatal sepsis with PROM were 0.85 (95% CI: 0.78–0.90), 0.87 (95% CI: 0.84–0.90), and 0.9410, respectively, which showed high stability.

### 3.6 Meta-regression analysis

Meta-regression analysis was conducted to investigate potential sources of heterogeneity (Table 2). The specimen and cut-off were not significant sources of heterogeneity (*P = *.6932 and 0.9540, respectively). However, the types of sepsis might be connected with the source of heterogeneity. The diagnostic accuracy of IL-6 in early onset neonatal sepsis was 16.6 times higher than that in early/late-onset neonatal sepsis (RDOR = 16.6, 95% CI: 1.31–209.61; *P = *.0351).

**Table 2:**
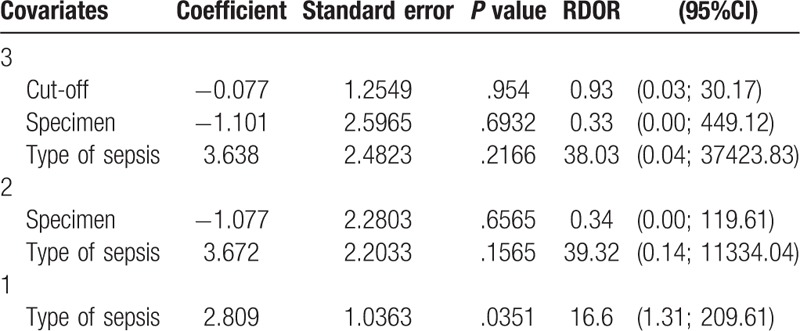
Meta-regression analysis of the effects of specimen, cut-off, and type of sepsis.

### 3.7 Subgroup analysis

Regarding the different specimens, a total of 580 samples in umbilical cord blood and 172 samples in neonatal peripheral blood were detected. The sensitivity and specificity in neonatal peripheral blood was higher than in umbilical cord blood (93% vs 83%, 97% vs 87%). Besides, the PLR of IL-6 in neonatal peripheral blood was higher than that in umbilical cord blood (27.86 vs 6.27). The NLR, DOR, and AUC are shown in Table 3.

**Table 3:**
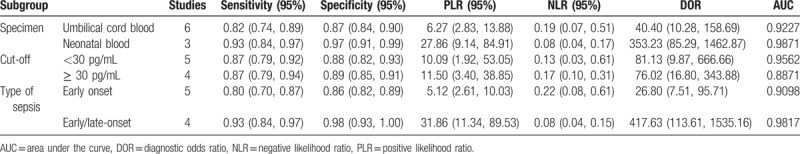
Subgroup analysis of study specimen, cut-off, and types of sepsis.

When the cut-off of IL-6 was more than 30 pg/mL, the specificity was the same with IL-6 <30 pg/mL (87%), and the sensitivity was comparable (89% and 88%). The PLR, NLR, DOR, and AUC are shown in Table 3.

Regarding types of sepsis, a total of 522 samples with early onset neonatal sepsis and 230 samples with early/late-onset neonatal sepsis were identified. The early/late-onset neonatal sepsis had a much higher sensitivity (93% vs 80%) and specificity (98% vs 86%) than early onset neonatal sepsis. Besides, the PLR of IL-6 in early/late-onset neonatal sepsis was higher than in early onset neonatal sepsis (31.84 vs 5.12). The NLR of IL-6 in early/late-onset neonatal sepsis was lower than in early-onset neonatal sepsis (0.08 vs 0.22). The DOR and AUC are shown in Table 3.

### 3.8 Publication bias

We used Deek's funnel plot asymmetry test to assess publication bias of the diagnostic tests. The results showed that the *t*-value obtained from the funnel plot was −2.18, and the *P*-value was .07, indicating no striking publication bias in this meta-analysis.

## 4 Discussion

Neonatal sepsis with PROM is a serious systemic disease. The reference standard for the diagnosis of neonatal sepsis with PROM is a positive blood culture, which is time-consuming, less sensitive, and has a high false-negative rate.^[6,16,17]^ Besides, neonatal sepsis does not occur in every PROM case; it is thus important to accurately predict neonatal sepsis with PROM, so that active management instead of expectant management can be undertaken to reduce the likelihood of the inadvertent use of antibiotics, cost of treatment, and overtreatment.^[7,33]^

Laboratory tests, such as CRP and PCT, are not sufficient for the early diagnosis of sepsis with PROM. The sensitivity and specificity of the CRP for the early diagnosis of neonatal sepsis with PROM were <60% and 80%, respectively.^[34,35]^ CRP often begins increasing after 12 to 24 hours of neonatal infection and peaks later, which is not conducive for the early diagnosis of neonatal sepsis with PROM. The sensitivity and specificity of PCT for the early diagnosis of neonatal sepsis with PROM are 53% and 45%, respectively. Besides, physiological PCT can increase from 1 to 10 ng/mL in normal neonates within 48 hours after delivery.^[35,36]^ IL-6 is a pleiotropic cytokine that is expressed by different cells in response to infections.^[22]^ Furthermore, IL-6 can be detected rapidly by flow cytometry with minimal blood volumes (0.05 mL). In recent years, IL-6 has been investigated for its validity in the early diagnosis of neonatal sepsis with PROM.^[2,7–13]^

A meta-analysis by Shahkar et al^[23]^ showed that the sensitivity and specificity of IL-6 for neonatal sepsis were 79% and 84% in neonates without PROM. However, in our study, we first conducted a systematic review and meta-analysis to investigate the early diagnostic value of IL-6 as a potential biomarker for neonatal sepsis with PROM. The pooled sensitivity of IL-6 for the diagnosis of neonatal sepsis with PROM was 0.87, the pooled specificity was 0.88, the missed diagnosis rate was 0.13, and the misdiagnosis rate was 0.12, which showed that the diagnostic efficiency was high. A PLR of 9.94 suggests that neonates with sepsis and PROM have a 9.94-fold higher chance of being IL-6-positive than neonates with PROM. This ratio suggests a potential role for IL-6 for neonatal sepsis with PROM. The DOR is a comprehensive evaluation of the diagnostic value of the index test; the pooled DOR was 61.81, which showed a good discriminating effect. The overall accuracy of IL-6 for the diagnosis of neonatal sepsis with PROM was favorable. These results indicate that IL-6 is a helpful biomarker for the early diagnosis of neonatal sepsis with PROM.

Different specimens may have different performance to IL-6. Generally speaking, umbilical cord blood and neonatal peripheral blood are neonatal blood. Although the specimens were not significant sources of heterogeneity (*P = *.6565), we found that the sensitivity and specificity in neonatal peripheral blood was higher than in umbilical cord blood. The small number of included studies might lead to this result. Therefore, further meta-analysis including more studies is needed to explain the reason.

With cut-off ≥30 pg/mL, the specificity was the same with cut-off <30 pg/mL. Although the cut-off range was 7.6 to 108.5 pg/mL, it was not a significant source of heterogeneity (*P = *.954) and showed similar diagnostic value with the overall diagnostic accuracy.

In this meta-analysis, meta-regression proved that the diagnostic accuracy of IL-6 test for predicting neonatal sepsis with PROM was affected by the types of sepsis (*P = *.0351). Furthermore, the results of the subgroup analysis showed that the AUC of early/late-onset neonatal sepsis and early-onset neonatal sepsis was 0.9817 and 0.9098, respectively. The type of sepsis was one cause of the heterogeneity, because of different pathogens and degrees in inflammatory response between early/late-onset neonatal sepsis and early-onset neonatal sepsis.

Our meta-analysis had several limitations. First, IL-6 tests are usually detected along with other cytokines, but we did not address reliability with respect to combined analyses of IL-6 and other cytokines. Second, some studies included individuals with neonatal sepsis and PROM after antibiotic treatment, while some were without antibiotic treatment. This may influence the diagnosis. Third, the heterogeneity was relatively high. Although different specimens and cut-off in meta-regression analysis are not significant sources of heterogeneity (*P* > .05), they can also increase the heterogeneity and reduce the generalizability of the overall performance. Third, some studies were rated as high risk, and this may have affected the pooled effects. Finally, publication bias was a concern. Because of the linguistic abilities of our team, only studies written in English or Chinese were included. The true accuracy of IL-6 tests for neonatal sepsis and PROM may be lower than our report.

## 5 Conclusions

The reference standard for the diagnosis of sepsis with PROM is a positive blood culture, which has various practical limitations. This meta-analysis shows that IL-6 might be a sensitive and specific diagnostic marker for the early diagnosis of neonatal sepsis with PROM. The diagnostic accuracy of IL-6 is not influenced by types of neonatal sepsis. To provide optimum diagnostic value, IL-6 should detect early onset and late-onset neonatal sepsis separately. Furthermore, large, multicentre, and prospective studies are warranted to support our findings.

## Author contributions

**Conceptualization:** Xia Qiu, Li Zhang.

**Data curation:** Xia Qiu, Li Zhang.

**Formal analysis:** Yu Tong, Yi Qu.

**Funding acquisition:** Huiqing Wang, Dezhi Mu.

**Investigation:** Xia Qiu, Li Zhang, Yu Tong, Huiqing Wang.

**Methodology:** Xia Qiu, Li Zhang.

**Project administration:** Xia Qiu, Li Zhang, Huiqing Wang, Dezhi Mu.

**Software:** Xia Qiu, Li Zhang, Yu Tong.

**Supervision:** Xia Qiu, Li Zhang, Huiqing Wang, Dezhi Mu.

**Validation:** Xia Qiu, Li Zhang, Dezhi Mu.

**Visualization:** Yu Tong, Yi Qu.

**Writing – original draft:** Xia Qiu, Li Zhang.

**Writing – review & editing:** Yi Qu, Dezhi Mu.

Dezhi Mu: 0000-0002-2599-7041.

Dezhi Mu orcid: 0000-0002-2599-7041.
